# SARS-CoV-2 induces transcriptional signatures in human lung epithelial cells that promote lung fibrosis

**DOI:** 10.1186/s12931-020-01445-6

**Published:** 2020-07-14

**Authors:** Jincheng Xu, Xiaoyue Xu, Lina Jiang, Kamal Dua, Philip M. Hansbro, Gang Liu

**Affiliations:** 1 School of Stomatology, Bengbu Medical College, Bengbu, 2033 Anhui China; 2 School of Public Health and Community Medicine, Faculty of Medicine, University of New South Wales, Kensington, 233000 NSW Australia; 3 Faculty of Health, University of Technology Sydney, Ultimo, NSW 2007 Australia; 4 Discipline of Pharmacy, Graduate School of Health, University of Technology Sydney, Ultimo, NSW 2007 Australia; 5 Centre for Inflammation, Centenary Institute, Camperdown, NSW 2050 Australia; 6 School of Life Science, Faculty of Science, University of Technology Sydney, Ultimo, NSW 2007 Australia

**Keywords:** Coronavirus, SARS-CoV-2, Angiotensin-converting enzyme 2, Lung fibrosis

## Abstract

**Background:**

Severe acute respiratory syndrome (SARS)-CoV-2-induced coronavirus disease-2019 (COVID-19) is a pandemic disease that affects > 2.8 million people worldwide, with numbers increasing dramatically daily. However, there is no specific treatment for COVID-19 and much remains unknown about this disease. Angiotensin-converting enzyme (ACE)2 is a cellular receptor of SARS-CoV-2. It is cleaved by type II transmembrane serine protease (TMPRSS)2 and disintegrin and metallopeptidase domain (ADAM)17 to assist viral entry into host cells. Clinically, SARS-CoV-2 infection may result in acute lung injury and lung fibrosis, but the underlying mechanisms of COVID-19 induced lung fibrosis are not fully understood.

**Methods:**

The networks of ACE2 and its interacting molecules were identified using bioinformatic methods. Their gene and protein expressions were measured in human epithelial cells after 24 h SARS-CoV-2 infection, or in existing datasets of lung fibrosis patients.

**Results:**

We confirmed the binding of SARS-CoV-2 and ACE2 by bioinformatic analysis. TMPRSS2, ADAM17, tissue inhibitor of metalloproteinase (TIMP)3, angiotensinogen (AGT), transformation growth factor beta (TGFB1), connective tissue growth factor (CTGF), vascular endothelial growth factor (VEGF) A and fibronectin (FN) were interacted with ACE2, and the mRNA and protein of these molecules were expressed in lung epithelial cells. SARS-CoV-2 infection increased *ACE2*, *TGFB1*, *CTGF* and *FN1* mRNA that were drivers of lung fibrosis. These changes were also found in lung tissues from lung fibrosis patients.

**Conclusions:**

Therefore, SARS-CoV-2 binds with ACE2 and activates fibrosis-related genes and processes to induce lung fibrosis.

## Background

Coronavirus (CoV) is a group of single-stranded RNA viruses and is a pathogen of the human respiratory system. CoV infection results in lethal respiratory diseases, including severe acute respiratory syndrome (SARS), middle east respiratory syndrome (MERS) and coronavirus disease-2019 (COVID-19). SARS induced by SARS-related coronavirus (SARS-CoV) affected 8096 patients from 2002 to 2003 with a fatality rate of 9.6% worldwide [1]. MERS-related coronavirus (MERS-CoV) affected 2519 cases with a high fatality of 34.4% [2]. As of 24th May 2020, a new strain of CoV, SARS-CoV-2 induced COVID-19 has leads to over 5.2 million cases in 188 countries, resulting in more than 337,000 deaths, and numbers substantially increase every day [3]. COVID-19 has become a public health emergency of international concern and designated a pandemic by WHO [3]. The lack of deep understanding of SARS-CoV-2 is hampering vaccine development.

The most severe sequela of pathogenic coronavirus infection-induced SARS is lung fibrosis that up to 45% of SARS patients develop lunf fibrosis after 3–6 months, and this potentially sets an important context for COVID-19 [4–7]. Lung fibrosis is characterised by excessive deposition of extracellular matrix (ECM) proteins, such as fibronectin (Fn). This results in impaired lung function and reduced gas exchange. Transforming growth factor beta (TGF-β) associated signalling pathway play important roles in lung fibrosis, but the role of this pathway in COVID-19 is unclear. A recent study shows that COVID-19 patients have a high risk of lung fibrosis [8]. Increasing studies show that COVID-19-induced acute respiratory distress syndrome (ARDS) results in diffused alveolar damages in lungs, and the cases of long-term ARDS leading to lung fibrosis are starting to be reported [9–13]. However, the links between SARS-CoV-2 and lung fibrosis remains unclear.

SARS-CoV and SARS-CoV-2 share approximately 76% amino acid sequence homology that lead to the similarities in their biological properties [14]. The spike (S) protein is a key structural component of CoV that binds to host cellular receptors that facilitates viral entry into target cells [15]. Angiotensin-converting enzyme 2 (ACE2) has been identified as a receptor of SARS-CoV-2 [4], which is cleaved by type II transmembrane serine protease (TMPRSS2) to augment virus entry into host cells [15]. ACE2 is also cleaved by a disintegrin and metallopeptidase domain (ADAM)17 of the host, which facilitates shedding of ACE2 into the extracellular space to bind with CoV [16]. However, it remains unclear how SARS-CoV-2 infection induces lung fibrosis.

In this study, we examined SARS-CoV-2 entry into target cells by binding with ACE2 after TMPRSS2 and ADAM17 cleavage. We found that human alveoli epithelial cells are the main target cells of SARS-CoV-2 rather than airway bronchial epithelial cells. SARS-CoV-2 infection alters gene expression, including tissue inhibitor of metalloproteinase (TIMP)3, angiotensinogen (AGT), TGFB1, connective tissue growth factor (CTGF), vascular endothelial growth factor (VEGF) A and FN1, and these changes are also observed in lung tissues from patients with lung fibrosis. SARS-CoV-2 infection likely activates TGF-β signalling, increases FN expression and results in lung fibrosis.

## Materials and methods

### Predicted SARS-CoV protein and ACE2 binding

Previous studies showed a conserved evolutionary relationship between SARS-CoV and SARS-CoV-2 [14]. The S protein of SARS-CoV-2 and its predicted receptor, ACE2 were identified based on a public database using p-hipster as previously described [17].

### The interaction network of ACE2 genes and proteins

Predicted gene/protein interactions were obtained from online databases using bioinformatics analysis. We used GeneMANIA (University of Toronto) to generate an interaction network of *ACE2* and related proteins [18]. Previous studies showed that TMPRSS2 cleaves ACE2 [19], and multiple gene queries were chosen in humans for searching the gene network of these two molecules. The predicted genes that interacted with *ACE2* and *TMPRSS2* were listed using cytoscape analysis (GeneMANIA cytosacpe plugin).

A connective network of ACE2 protein and its functional interactions were obtained using STRING version 11.0 (ELIXIR Infrastructure) as previously described [20]. Briefly, ACE2 and TMPRSS2 were used in main searching list name and organism was *Homo sapiens*. We selected textmining, experiments, databases and co-expression as active interaction sources. High confidence was used as the interaction score and the disconnected nodes in the network were hidden to simplify the display.

### Genotype-tissue expression (GTEx) pilot analysis

The GTEx project is an RNA-sequencing database of gene expression in different tissues [21]. It links regulatory expression quantitative trait loci (eQTL) variants (gene expression) to tissues. A network of *ACE2*, *TMPRSS2*, *ADAM17*, *TIMP3*, *AGT*, *TGFB1*, *VEGFA*, *CTGF* and *FN1* genes across all human tissues were generated using GTEx Portal as previously described [22].

### Protein detection in lungs

Representative images of ACE2, TMPRSS2, ADAM17, AGT, TGFB1, VEGFA, CTGF and FN proteins in human lung tissues were obtained from the Human Protein Atlas database as previously described [23]. The Tissue Atlas and Pathology Atlas database (version 19.3) was mined for the expression and localization of these proteins in the lung tissues by immunohistochemistry, and representative images were taken to show the localisation of the target proteins in lung tissues [24].

### Single cell analysis of human lung datasets

We analysed the expressions of *ACE2*, *TMPRSS2*, *ADAM17*, *TIMP3*, *AGT*, *TGFB1*, *VEGFA*, *CTGF* and *FN1* in different lung cell populations using previously published human single cell RNA-sequencing datasets. All datasets were explored in the UCSC cell browser to identify the cellular sources of those genes in the airways or lung tissues.

In the first dataset [25], human bronchial epithelial cells (HBECs) were obtained from endobronchial lining fluid by invasive bronchoscopy microscampling (*n* = 4), and lung samples (*n* = 12) were obtained by surgical intervention. Endobronchial lining fluid was collected from non-involved segment from the contralateral lungs of patients with lung cancer, and HBECs were isolated and grew in culture media [25]. Lung tissues were obtained from lung cancer patients, and normal lung tissues was distant from the tumour area [25]. These samples were snap-frozen by liquid nitrogen without direct touch and stored at − 80 °C. RNA-sequencing was performed using 10X Genomics Chromium platform of IIIumina HiSeq4000. In the second dataset [26], single cells were isolated from cryobiopsy samples from one idiopathic pulmonary fibrosis (IPF) patient. In the third dataset, single cell samples were obtained from lung biopsies from donors with healthy lungs, but were dead with other diseases or accident (*n* = 8), including stroke (one patient), intracranial haemorrhage (three patients), anoxic brain injury (three patients) and head trauma from gunshot wound (one patient) and patients with pulmonary fibrosis (*n* = 8), including IPF (four patients), interstitial lung diseases (ILD, three patients) and hypersensitivity pneumonitis (one patient) [26].

Cells were clustered using a graph-based shared nearest neighbor clustering approach and graphs were visualised using a t-distributed Stochastic Neighbor Embedding (tSNE) plot to identify the main cellular source of those genes in the airways or lungs.

### Gene expressions in human epithelial cells treated with SARS-CoV-2

The gene expressions of *ACE2*, *TMPRSS2*, *ADAM17*, *TIMP3*, *AGT*, *TGFB1*, *VEGFA*, *CTGF* and *FN1* were from an existing RNA-sequencing dataset [27] through Gene Expression Omnibus (GEO) database. The data were analyzed using Bioconductor in R (Bioconductor) as previously described [28–30]. Briefly, in the GSE147507 dataset [27], human adenocarcinoma alveolar basal epithelial (A549, 1 × 10^6^) cells and HBECs (1 × 10^5^) were infected with SARS-CoV-2 (deposited by the Centre for Disease Control and Prevention and obtained through BEI Resources) or media controls for 24 h and total RNA was extracted by TRIzol Reagent (ThermoFisher). RNA-seq libraries of polyadenylated RNA were prepared using the TruSeq RNA library Prep Kit V2 (Illumina) and RNA-seq libraries for total ribosomal RNA-depleted RNA were prepared using the TruSeq Stranded Total RNA library Prep Gold (Illumina).

### Gene expression in human lung fibrosis datasets

We analysed the gene expression of *ACE2*, *TMPRSS2*, *ADAM17*, *TIMP3*, *AGT*, *TGFB1*, *VEGFA*, *CTGF* and *FN1* in lung samples from pre-existing gene microarray datasets.

In GSE2052 dataset [31–33], lung tissues were obtained from healthy controls (*n* = 11) and IPF patients (*n* = 13). DNA was isolated from lung histology for gene array analysis and data was profiled by an Amersham Biosciences Codelink uniset human bioarray.

In the GSE10667 dataset [34–36], lung tissues were from lung healthy controls (*n* = 15), ILD patients with usual interstitial pneumonia (UIP) histopathologic pattern but not IPF (other ILD, *n* = 23) or IPF patients (*n* = 8). Samples were obtained from surgical remnants of biopsies or lungs explanted from patients with IPF who underwent lung transplant. Control normal lung tissues obtained from the disease-free margins with normal histology of lung cancer resection specimens. Gene expression was profiled by Agilent-014850 Whole Human Genome Microarray 4x44K G4112F.

The Benjamini-Hochberg method for adjusted *P* value/false discovery rate (FDR) was used to analyse differences between groups. Statistical significance was set at FDR < 0.05. Target gene expression was calculated as log_2_ intensity robust multi-array average signals (Log_2_ transformed intensity value) [37].

### Statistical analysis

Results are presented as mean ± standard error of the mean (SEM). Unpaired student *t*-Tests were used to compare two groups in existing dataset analysis. A one-way analysis of variance (ANOVA) with Bonferroni comparisons was used to compare between multiple groups [38]. All statistical analyses were performed using GraphPad Prism Software (San Diego, CA, USA) as previously described [39].

## Results

### Network of ACE2 and selected interacting factors in SARAS-CoV-2 infection

**Fig. 1:**
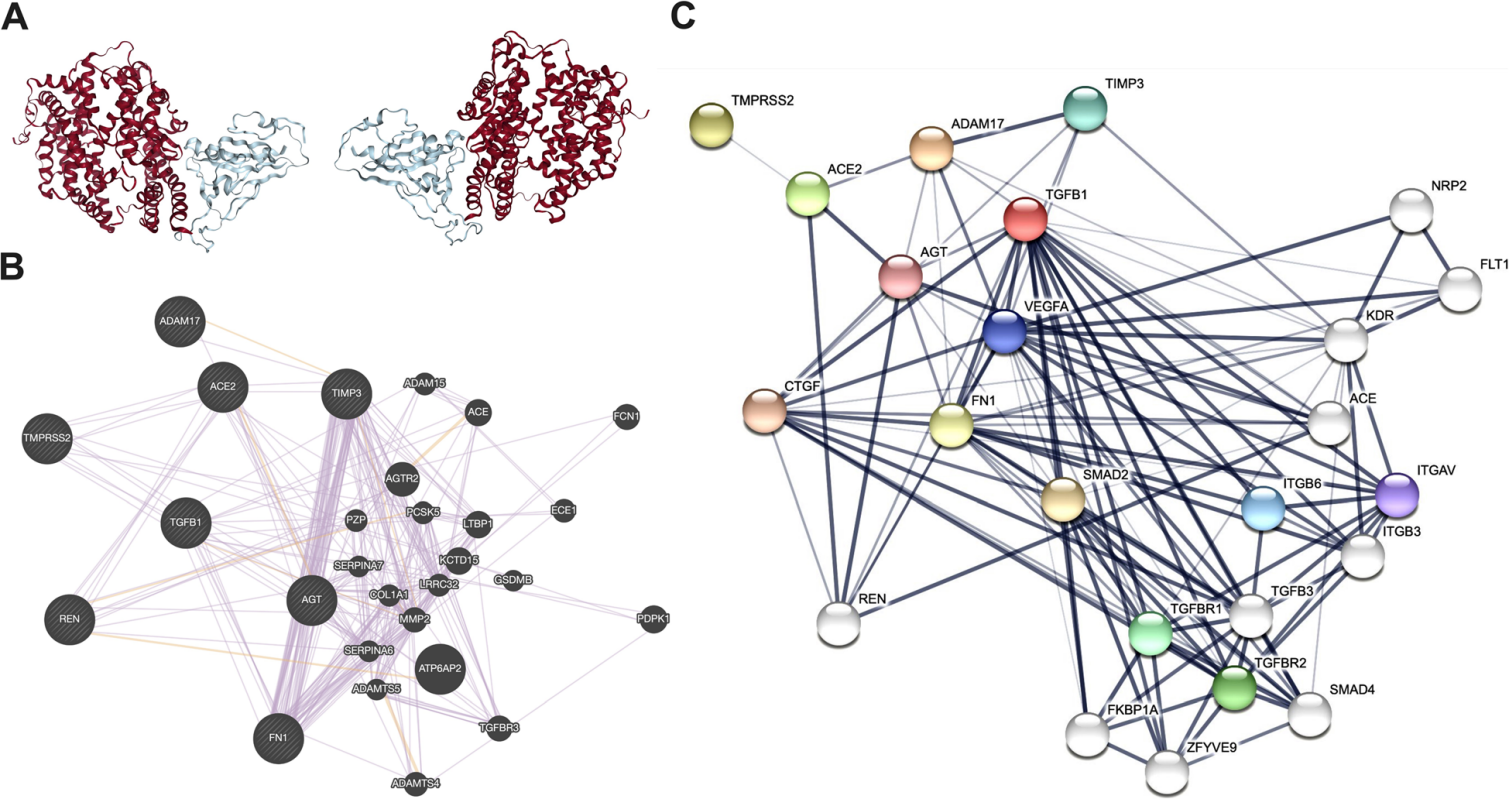
In silico modelling showing that SARS-CoV-2 may bind to ACE2 and associates with interacting molecules. **a** The spike (S) protein of SARS-CoV is predicted to bind with ACE2 protein and represented front and back images of protein structure using p-hipster. The predicted network of ACE2 gene (**b**) and protein (**c**) interacting molecules identified using bioinformatic analysis

We confirmed that the main S protein of SARS-CoV (grey color) bound to ACE2 protein (red color) using p-hipster analysis (Fig. 1a), which is consistent with previous studies [40]. ADAM17 and TMPRSS2 are the main enzymes that cleave ACE2 and promote SARS-CoV-2 entry into host cells [19]. To understand the process of SARS-CoV-2 binding, we identified the network of *ACE2* and its interacting genes using Gene MANIA [18]. *TMPRSS2*, *ADAM17*, *TIMP3*, *AGT*, *TGFB1*, *FN1* and renin (*REN*) were indicated to interact with *ACE2* (Fig. 1b). We then performed a bioinformatic analysis at the protein level of these molecules to show their protein-protein binding relationship using STRING (Fig. 1c). We found that TMPRSS2, ADAM17, TIMP3, AGT, TGFB1 and FN were the main proteins that interact with ACE2 after binding with SARS-CoV-2. We excluded *REN* in the following analysis as this molecule was not highlighted by protein interaction analysis.

### ACE2 and its interacting proteins were found in lung

**Fig. 2:**
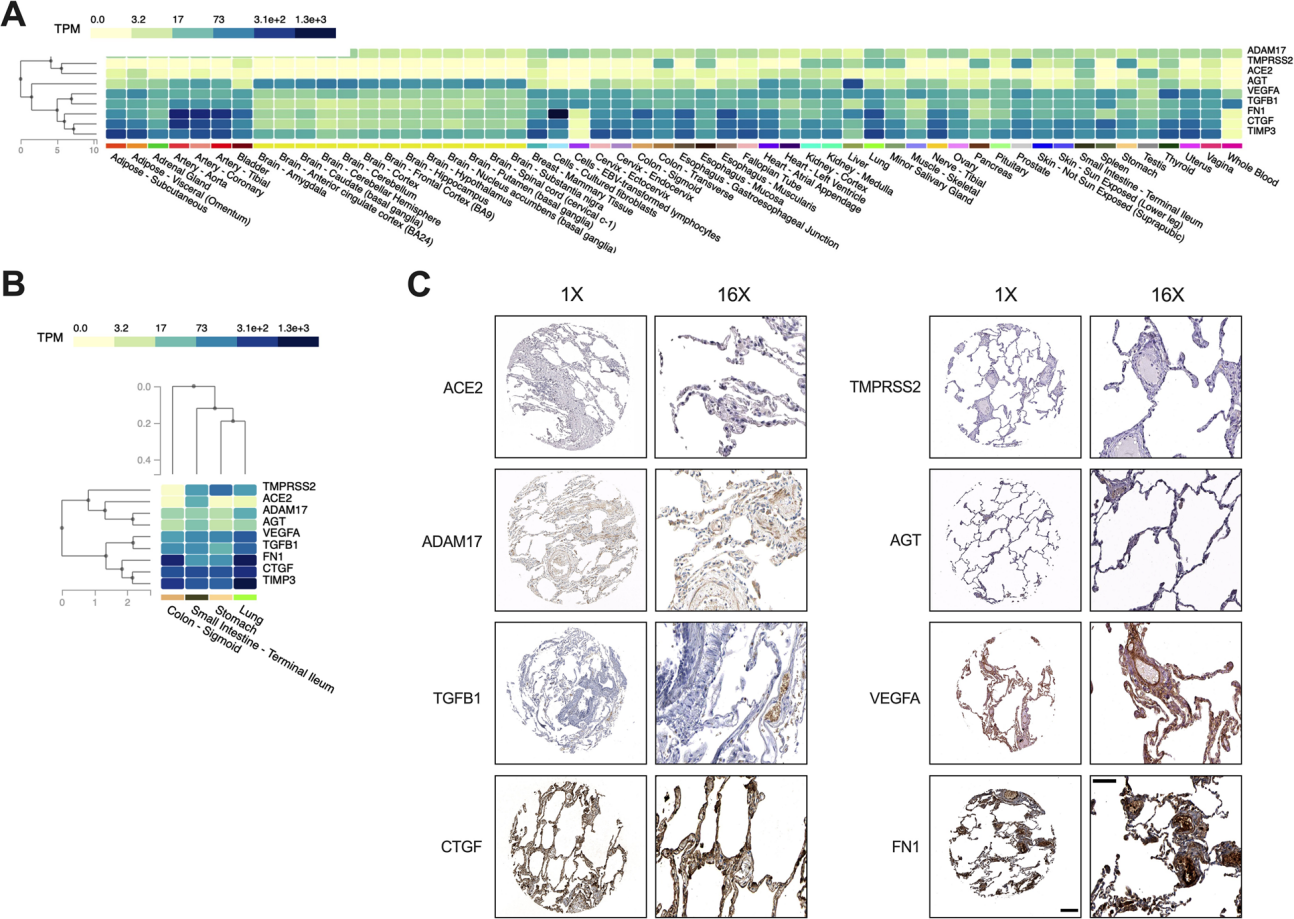
mRNA transcripts and protein levels of ACE2 and its interacting molecules in human in the lungs and gut. **a** mRNA expression of *ACE2*, *TMPRSS2*. *ADAM17*, *TIMP3*, *TGFB1*, *CTGF*. *VEGFA* and *FN1* in all human organs and **b** in the lung and gastrointestinal tract using GTEx Portal. **c** ACE2, TMPRSS2, ADAM17, AGT, TGFB1, VEGFA, CRGF and FN proteins in human lung using immunohistochemistry from Pathology Atlas database (1x scale bar is 200 μm, 16x scale bar is 100 μm)

To further investigate the role of ACE2 and its interacting proteins in SARS-CoV-2 infection/COVID-19, the expression of *ACE2* gene in different organs were identified using GTEx portal (Fig. 2a). *ACE2* mRNA expression in the lung was lower than levels in the small intestine (Fig. 2b). Most of the mRNAs of selected *ACE2* interacting molecules were highly expressed in human lungs, including *ADAM17*, *TIMP3*, *TGFB1*, *VEGFA*, *CTGF* and *FN1*. To indicate that the expression of these genes was converted into protein, we identified which proteins are high abundant in the lungs and assessed the cellular sources of their proteins in human lung using a Human Protein Atlas database [23]. All of these proteins were found in lung epithelial cells based on their cellular morphology. While ACE2 and TMPRSS2 proteins occurred at a low level ADAM17, AGT and TGFB1 had a moderate level in normal lung tissues (Fig. 2c). VEGFA, CTGF and FN1 proteins were highly expressed in lung tissues, and TIMP3 was not present in this protein database.

### ACE2 and its interacting factors were expressed in lung epithelial cells

**Fig. 3:**
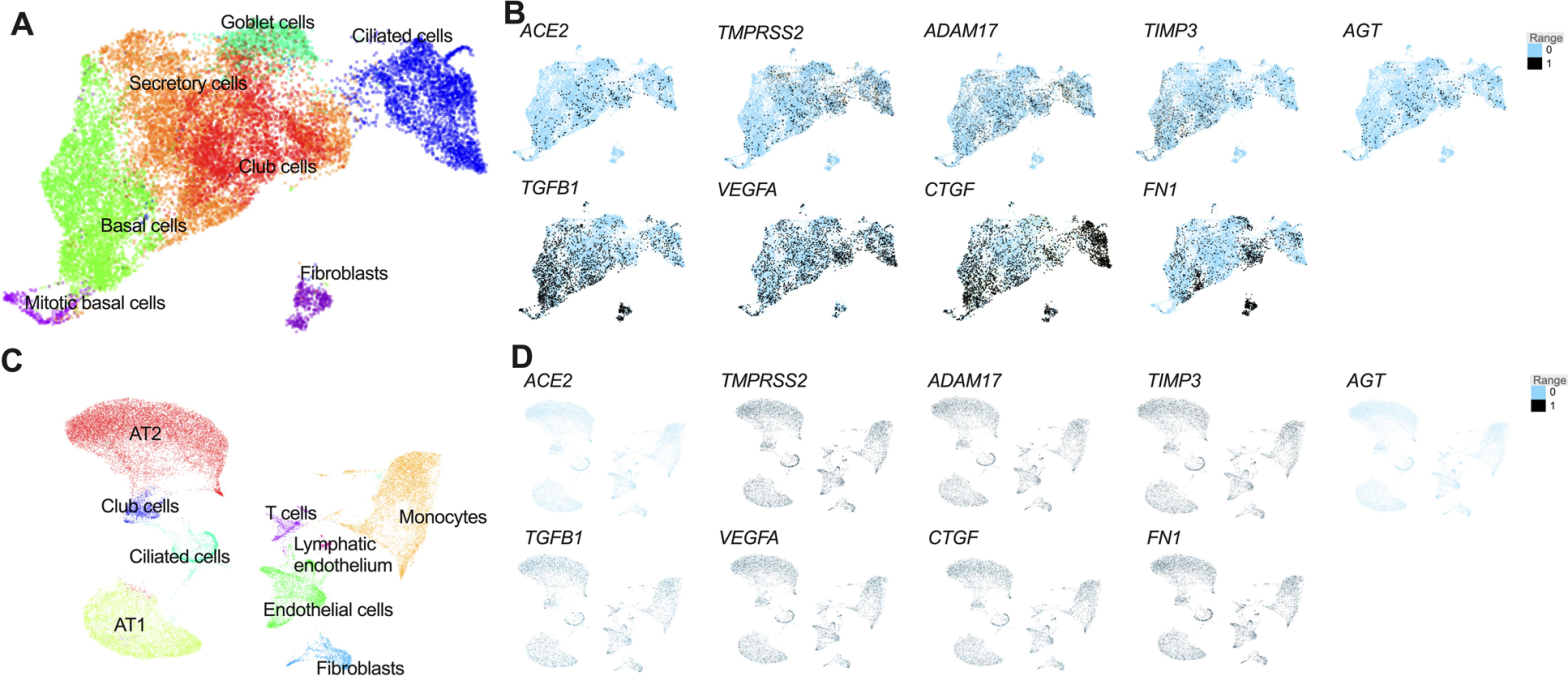
Single cell RNA-sequencing analysis of *ACE2*, *TMPRSS2*. *ADAM17*, *TIMP3*, *AGT*, *TGFB1*, *CTGF*. *VEGFA* and *FN1* mRNAs in human airways and lungs. **a** Single cells were isolated from human bronchial biopsy (*n* = 4) and subjected to RNA-sequencing. Cells were clustered using a graph-based shared nearest neighbour clustering approach and visualised using a t-distributed Stochastic Neighbor Embedding (tSNE) plot from UCSC cell browser. **b** mRNA expression of *ACE2*, *TMPRSS2*. *ADAM17*, *TIMP3*, *TGFB1*, *CTGF*, *VEGFA* and *FN1* in different cells from human airways. **c** Single cells were isolated from human lungs (*n* = 12) and the cells were clustered using tSNE plot from UCSC cell browser. **d** mRNA expression of *ACE2*, *TMPRSS2*. *ADAM17*, *TIMP3*, *TGFB1*, *CTGF*, *VEGFA* and *FN1*in different cells from human lungs

There were two main types of epithelial cells in the lung, airway and alveolar epithelial cells [41]. To further examine the specific cellular source of ACE2 and interacting factors in human airways, cells were collected from human bronchial biopsies and single cell RNA-sequencing analysis was performed (Fig. 3a). The mRNA expression of *ACE2* and all of the selected interacting factors, including *TMPRSS2*, *ADAM17*, *TIMP3*, *AGT*, *TGFB1*, *VEGFA*, *CTGF* and *FN1* were detectable in bronchial epithelial cells, while *TGFB1*, *VEGFA*, *CTGF* and *FN1* were also found in airway fibroblasts (Fig. 3b). We then explored the cellular sources of these molecule in lung tissues, in particular the parenchyma (Fig. 3c). *ACE2* and *AGT* mRNAs were found in type 1 (AT1) and type 2 alveolar epithelial (AT2) cells, but their levels were lower than other selected mRNAs, *TMPRSS2*, *ADAM17*, *TIMP3*, *TGFB1*, *VEGFA*, *CTGF* and *FN1* (Fig. 3d).

### SARS-CoV-2 infection affects ACE2 and its selected interacting factors in alveolar but not bronchial epithelial cells

**Fig. 4:**
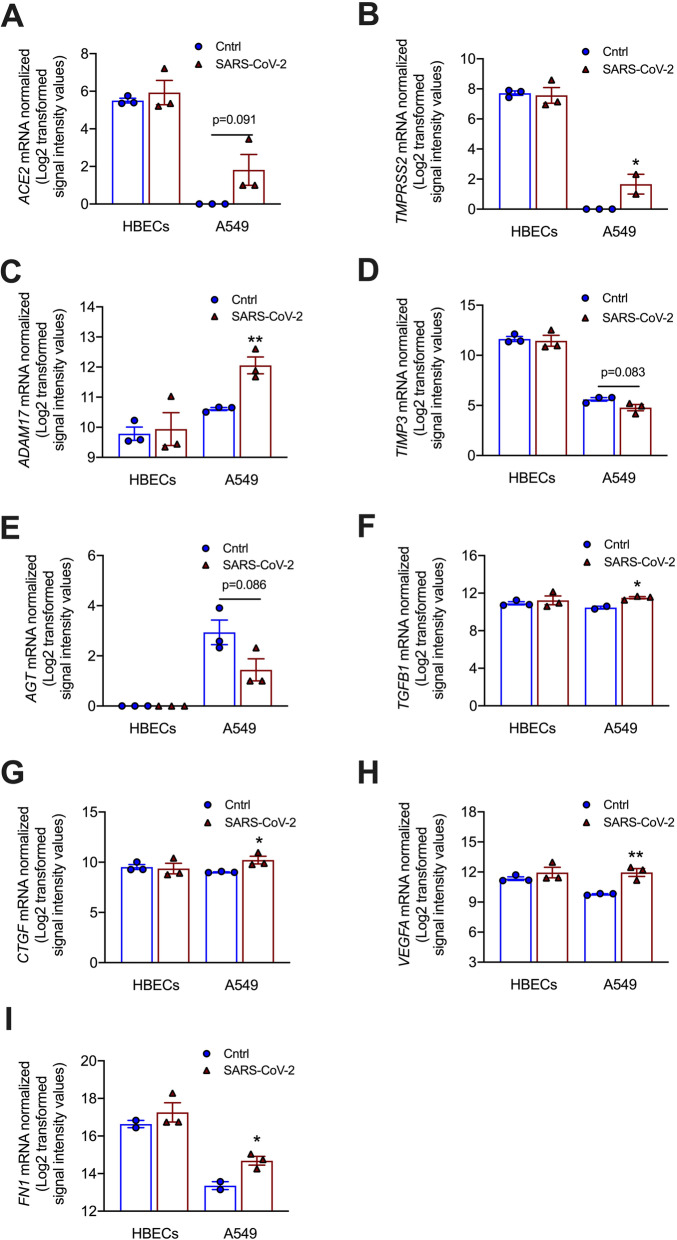
SARS-CoV-2 infection induces Ace2 gene and its interacting factors in human alveolar but not bronchial epithelial cells. Human bronchial epithelial cells (HBEC) and alveolar epithelial (A549) cells were infected with SARS-CoV-2 for 24 h, and control cells received medium only. The mRNA expression of *ACE2* (**a**), *TMPRSS2* (**b**), *ADAM17* (**c**), *TIMP3* (**d**), *AGF* (**e**), *TGFB1* (**f**), *CTGF* (**g**), *VEGFA* (**h**) and *FN1* (**i**) in HBECs and alveolar epithelia cells. *n* = 2–3, *P < 0.05, **P < 0.01 compared to control cells

HBECs and alveolar epithelial (A549) cells were inoculated with SARS-CoV-2 for 24 h and the mRNA expression of *ACE2* and selected interacting factors were assessed based on an existing RNA-seq dataset (GSE147507). *ACE2* mRNA expression had a non-significant trend to increase (*p* = 0.091) in alveolar epithelial cells with SARS-CoV-2 infection compared to sham-infected controls (Fig. 4a). Infection significantly increased the expression of *ACE2*, *TMPRSS2*, *ADAM17*, *TGFB1*, *CTGF*, *VEGFA* and *FN1* but resulted in trends towards decreases in *TIMP3* (*p* = 0.083) and *AGF* (*p* = 0.086) in A549 cells compared to control cells (Fig. 4b–i). However, the mRNA expression of *ACE2* and interacting factors were not changed in HBECs with infection.

### Alveolar epithelial cells are major cellular sources of ACE2 and interacting factors in lung fibrosis

**Fig. 5:**
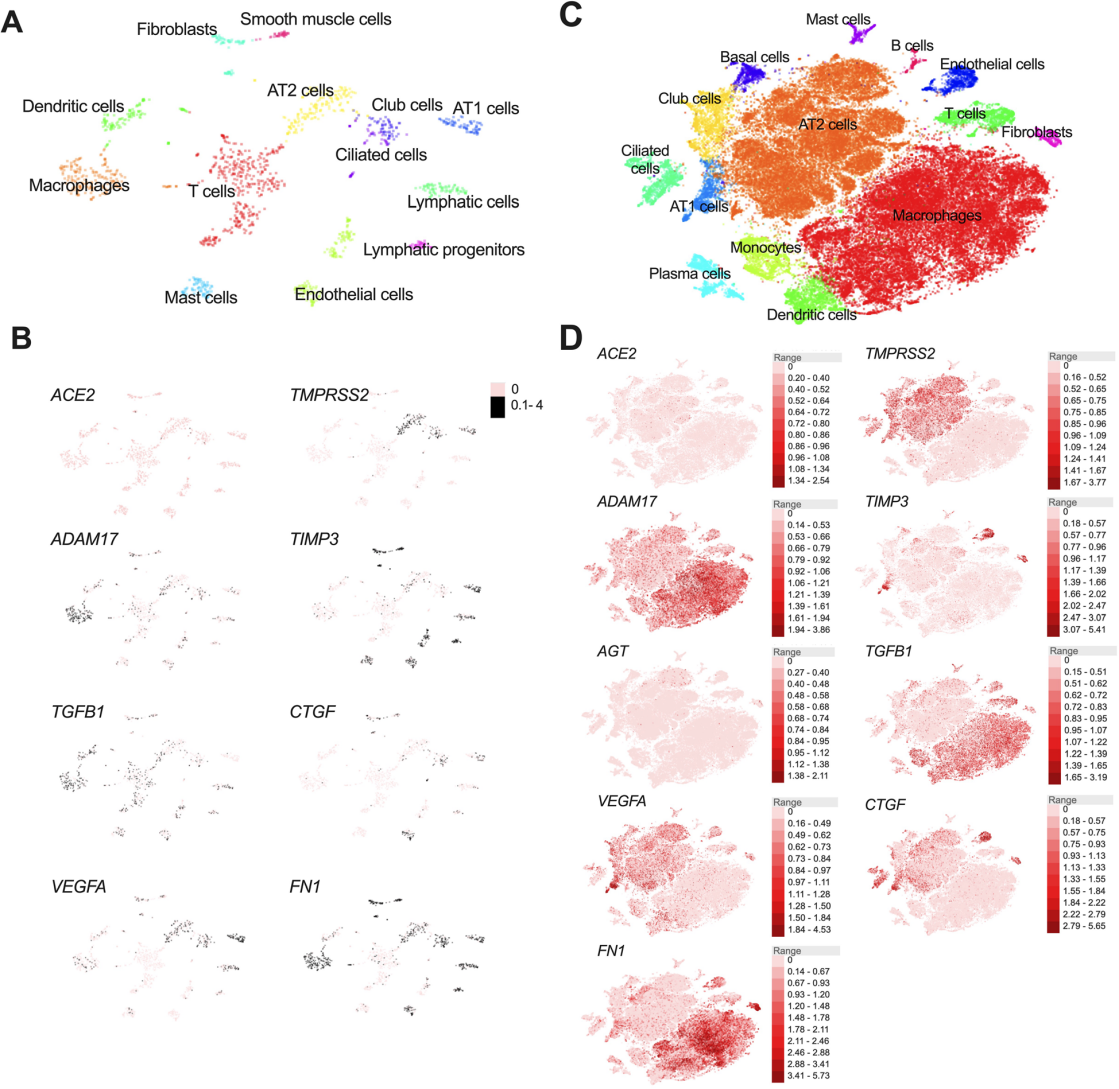
Single cell RNA-sequencing analysis of *ACE2*, *TMPRSS2*. *ADAM17*, *TIMP3*, *TGFB1*, *CTGF*, *VEGFA* and *FN1* mRNA in human lungs from patients with idiopathic pulmonary fibrosis (IPF). **a** Single cells were isolated from cryobiopsy samples in one IPF patient. They were subjected to RNA-sequencing and data clustered using a graph-based shared nearest neighbour clustering approach and visualised using a t-distributed Stochastic Neighbor Embedding (tSNE) plot from UCSC cell browser. **b** mRNA expression of *ACE2*, *TMPRSS2*, *ADAM17*, *TIMP3*, *TGFB1*, *CTGF*, *VEGFA* and *FN1* in different cells from human airways. **c** Single cells were isolated from human lungs from IPF patients (*n* = 8), subjected to RNA-sequencing and data clustered using tSNE plot from UCSC cell browser. **d** mRNA expression of *ACE2*, *TMPRSS2*, *ADAM17*, *TIMP3*, *AGT*, *TGFB1*, *CTGF, VEGFA* and *FN1* in different cells from human lungs

To understand the link between SARS-CoV-2 and lung fibrosis, we identified the cellular source of *ACE2* and interacting factors in cryobiopsy samples from one IPF patient using an existing single cell RNA-sequencing dataset (Fig. 5a). The mRNAs of *ACE2*, *TMPRSS2*, *ADAM17*, *TIMP3*, *TGFB1*, *CTGF*. *VEGFA* and *FN1* were found in alveolar epithelial cells (Fig. *5*b). To further confirm alveolar epithelial cells were the cellular sources of the interacting factors, we analysed another single cell RNA-sequencing dataset from the lung tissues of eight IPF patients (Fig. 5c). *ACE2* mRNA was mainly found in alveolar epithelial cells, but its level was low in lung tissues (Fig. 5d). The mRNAs of *ACE2* interacting factors were found in epithelial cells, confirming our previous data (Fig. 2). *TMPRSS2* and *VEGFA* mRNAs were mainly found in all types of epithelial cells, but *ADAM17*, *TGFB1* and *FN1* mRNAs were also detected in macrophages and fibroblasts.

### *ACE2* and fibrotic related genes were also found in lung fibrosis patients

**Fig. 6:**
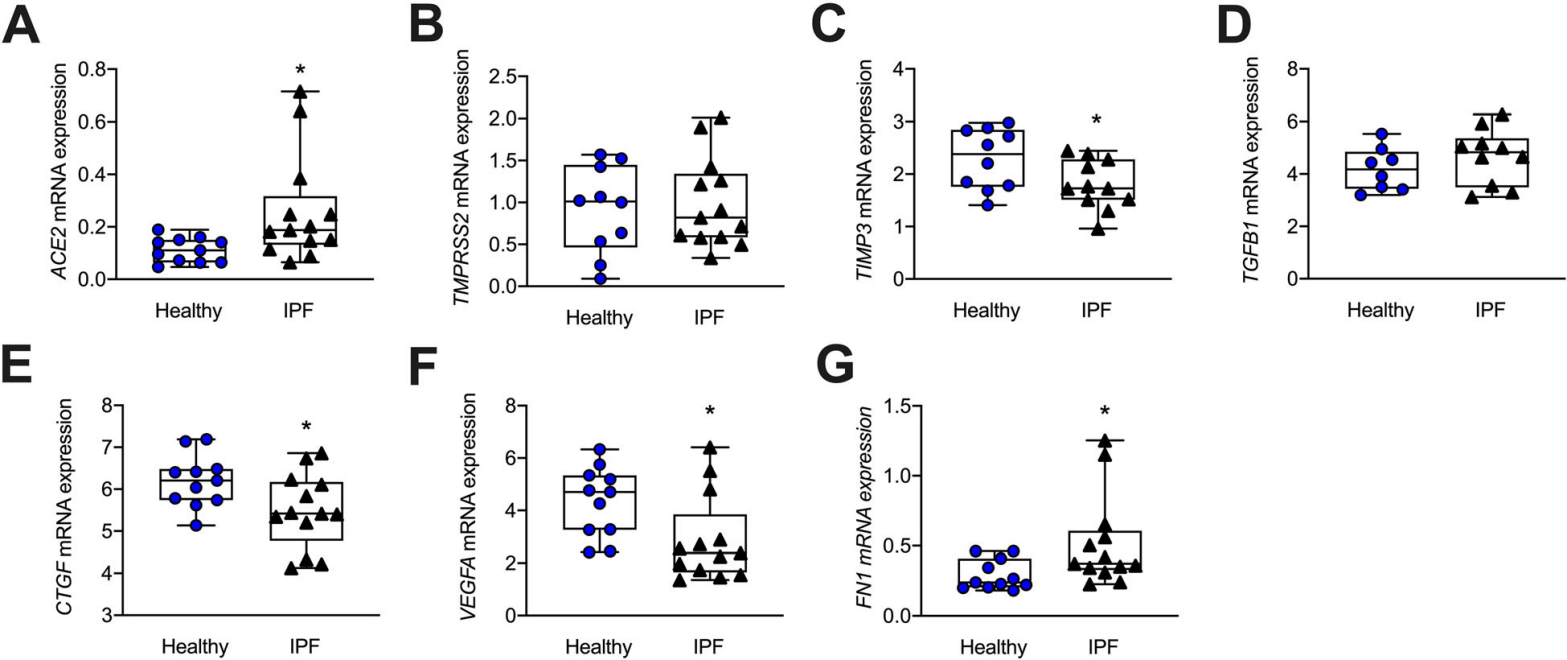
mRNA expression of *ACE2* and selected interacting factors in lung tissues from IPF patients. mRNA expression of *ACE2* (**a**), *TMPRSS2* (**b**), *TIMP3* (**c**), *TGFB1* (**d**), *CTGF* (**e**). *VEGFA* (**f**) and *FN1* (**g**) in lung tissues from IPF patients (*n* = 13) and lung healthy control (*n* = 11) were extracted from an existing microarray dataset (GSE2052). *P < 0.05 compared to lung healthy controls

To understand how SARS-CoV-2 infection may induce lung fibrosis, we measured the mRNA expression of *ACE2* and interaction factors in pre-existing microarray datasets from histological tissues from IPF patients and healthy controls [31–33]. IPF is a severe form of lung fibrosis [42], but it is unclear what causes of this disease. *ACE2* mRNA expression was significantly increased in lung tissues from IPF patients compared to controls (Fig. 6a). *TMPRSS2* mRNA was not changed (Fig. 6b), but *TIMP3* mRNA levels were decreased in IPF patients (Fig. 6c). *TGFB1* mRNA expression was not statistical different but there was a trend to an increase in IPF patients (Fig. 6d). Both *CTGF* and *VEGFA* mRNAs were decreased but *FN1* mRNA was significantly increased in IPF patients (Fig. 6e–g). *ADAM17* and *AGT* mRNAs were not detectable in this dataset.

**Fig. 7:**
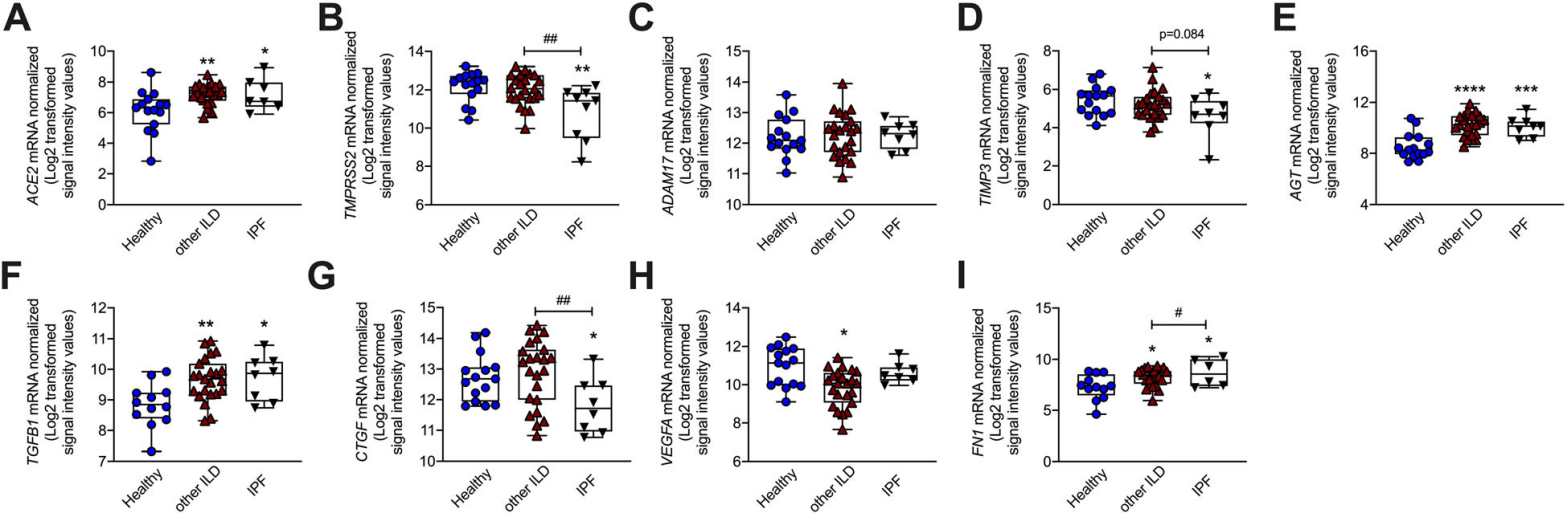
mRNA expression of *ACE2* and selected interacting factors in lung tissues from IPF and other ILD patients. mRNA expression of *ACE2* (**a**), *TMPRSS2* (**b**), *ADAM17* (**c**), *TIMP3* (**d**), *AGF* (**e**), *TGFB1* (**f**), *CTGF* (**g**). *VEGFA* (**h**) and *FN1* (**i**) in lung tissues from IPF (*n* = 8), other ILD patients (*n* = 23) and lung healthy control (*n* = 15) were extracted from an existing microarray dataset (GSE10667). *P < 0.05, **P < 0.01, ***P < 0.001, ****P < 0.0001 compared to lung healthy controls. #*P* < 0.05, ##*P* < 0.01 compared to other ILD patients

To further identify possible links between COVID-19 and lung fibrosis, we analysed the mRNA expressions of *ACE2* and interaction factors in a different dataset where RNA was isolated from fresh lung tissues from IPF patients and lung healthy controls [34, 35]. We also included a group of other ILD patients with UIP pattern, which is another lung fibrosis disease with known causes compared to IPF [41]. *ACE2* mRNA was increased in lung tissues from both IPF and other ILD patients compared to controls (Fig. 7a). *TMPRSS2* mRNA was significantly reduced in lung tissues from IPF patients compared to other ILD patients and control (Fig. 7b). *ADAM17* mRNA was not changed between all groups (Fig. 7c), and there was a trend to reduced *TIMP3* mRNA (*p* = 0.084) in IPF patients compared to other groups (Fig. 7d). *AGF* and *TGFB1* mRNAs were increased in the patients with both forms of lung fibrosis compared to controls (Fig. 7e and f), but *CTGF* mRNA was decreased in IPF patients compared to other groups (Fig. 7g). *VEGFA* mRNA was decreased in other ILD patients compared to other groups (Fig. 7h). *FN1* mRNA was increased in lung tissues from IPF patients compared to other groups, was also increased in other ILD patients compared to controls (Fig. 7i).

## Discussion

COVID-19 is a pandemic disease that is induced by SARS-CoV-2. As of 24th May, it has affected more than 5.2 million people across the world causing 337,000 deaths. Studies demonstrate that SARS-CoV-2 infection may result in the similar effects as SARS-CoV due to the similarity of their sequence [14]. One of the major consequences of SARS is that patients develop lung fibrosis as a major sequela. Increasing studies show that COVID-19 patients have lung fibrosis [9–12], however it remains unknown how SARS-CoV-2 infection induces this. ACE2 is a cellular receptor of SARS-CoV-2, and we have confirmed the potential binding relationship of ACE2 and SARS-CoV-2 using bioinformatic analysis in the current study. In addition, we also show that SARS-CoV-2 infection associates with increases of fibrosis-related gene transcription that induces lung fibrosis.

The baseline level of *ACE2* mRNA expression is very low in lungs compared to other organs. It is increased in alveolar epithelial cells after SARS-CoV-2 infection, indicating a positive correlation of ACE2 and SARS-CoV-2 infection. We have found that *ACE2* mRNA expression is mainly found in gastrointestinal (GI) tract and the small intestine has the highest level of *ACE2* levels compared to other organs in this study. Diarrhea is one of major symptoms of COVID-19 and high numbers of ACE2 positive small intestine cells occur in COVID-19 patients [43]. This indicates that SARS-CoV-2 also may also affect the GI tract through the ACE2 receptor. It remains unclear how SARS-CoV-2 reaches the GI tract in COVID-19 patients. Possible routes are through infected food [44] or transmission from the lung to the GI tract via the lung-gut axis [45–47]. Live SARS-CoV-2 was detected in stool samples from patients who had respiratory issues but not diarrhea [48], suggesting SARS-CoV-2 infection occurs through lung-gut axis. On the other hand, a recent study showed that three children had positive SARS-CoV-2 tests in their stools, but negative results in their throat swab samples, indicating the virus enters these patients via oral infection [49]. The infection may also transmit from the gut to the lung [50], causing a secondary infection [39]. Respiratory and digestive systems are the two major pathways that SARS-CoV-2 enters the body. Thus, it has been recommended that routine stool testing should be performed in potential COVID-19 patients even after viral RNA clearance in their respiratory system [51].

There is a high chance that COVID-19 patients potentially develop lung fibrosis, but how infection leads to fibrosis remains unclear. TGF-β is a cytokine that promotes the development of fibrosis. Active TGF-β regulates the level of ECM proteins, which are major factors involved in tissue remodelling and fibrosis [42]. CTGF is another cytokine involved in the remodelling process and the induction of lung fibrosis [52]. We find that *TGFB1* and *CTGF* mRNA transcripts are significantly increased in alveolar epithelial cells after SARS-CoV-2 infection. FN is a major ECM protein that has critical roles in tissue remodelling and fibrosis [53]. Our previous studies showed that increased FN deposition is linked with lung fibrosis [42], and we show in the current study that increased *FN1* mRNA transcripts are present in lung tissues from lung fibrosis patients. Inhibiting a main functional domain of the *FN1* gene inhibits fibrosis features in an in vivo model of lung fibrosis [54]. In this study, we found that SARS-CoV-2 infection induced *FN1* gene expression in alveolar epithelial cells, indicating the early induction of fibrotic processes and how the virus may be driving this.

ACE2 is cleaved by ADAM17 and/or TMPRSS2 before SARS-CoV-2 binds, and the cleavage of the receptor facilitates virus entry into host cells [16]. These events may be self-promoting and the mRNA expression of *TMPRSS2* and *ADAM17* are increased in alveolar epithelial cells after SARS-CoV-2 infection. The enzyme activity of ADAM17 is inhibited and regulated by TIMP3 [55], but SARS-CoV-2 reduces *TIMP3* mRNA expression in alveolar epithelial cells, that likely promotes greater ADAM17 activity in COVID-19 patients. TMPRSS2 and ADAM17 may compete for ACE2 cleavage, and processing by TMPRSS2 promotes more virus entry than that of ADAM17 [19]. Thus, increased activity of these enzymes after SARS-CoV-2 infection may contribute lung fibrosis but this needs to be proven clinical and experimental studies.

Bronchial epithelial cells mount the initial response SARS-CoV-2, however we show that *ACE2* mRNA levels are not changed in HBECs after infection compared to sham-infected controls. HBECs mount little response to infection compared to alveolar epithelial cells, and induces pneumonia, suggesting that SARS-CoV-2 infection directly induces disorders in parenchyma, including lung fibrosis. HBECs may respond to a higher inoculum of SARS-CoV-2 or in a shorter timeframe that require further experiment.

Abnormal tissue remodelling results in lung fibrosis and this process is currently irreversible [56]. Pulmonary fibrosis patients have only an average 2–3 years survival of the after they have been confirmed with this lethal disease [57]. Many lung fibrosis patients do not have major or previous symptoms, but have late stage lung fibrosis upon diagnosis. Thus, the most responsive treatment time may be missed. Early diagnosis is now considered critical but is a major challenge. Since COVID-19 patients may develop lung fibrosis [9–12], early prevention and intervention may significantly reduce the number of lung fibrosis patients-induced by SARS-CoV-2 infection.

## Conclusion

Taken together, we have shown links between SARS-CoV-2 binding with ACE2 and TGF-β and CTGF. This process may induce ECM products, such as FN in alveolar epithelial cells, and may result in lung fibrosis in COVID-19 patients. Thus, a routine analysis and early prevention, diagnosis and treatment of lung fibrosis may be beneficial for COVID-19 patients.

## Data Availability

The datasets generated and analysed during the current study are available in the Gene expression omnibus [https://www.ncbi.nlm.nih.gov/geo/] and UCSC bell browser [https://cells.ucsc.edu/#] repositories.

## Abbreviations

ACE: Angiotensin-converting enzyme
ADAM: A disintegrin and metallopeptidase domain
AGT: Angiotensinogen
AT1: Type 1 alveolar epithelial
AT2: Type 2 alveolar epithelial
CoV: Coronavirus (CoV)
COVID-19: Coronavirus disease-2019
CTGF: Connective tissue growth factor
FN: Fibronectin
GEO: Gene expression omnibus
eQTL: Expression quantitative trait loci
GI: Gastrointestinal
HBECs: Human bronchial epithelial cells
ILD: Interstitial lung disease
IPF: Idiopathic pulmonary fibrosis
MERS: Middle east respiratory syndrome
REN: Renin
SARS: Severe acute respiratory syndrome
SARS-CoV: SARS-related coronavirus
TGFB1: Transformation growth factor beta
TIMP: Tissue inhibitor of metalloproteinase
TMPRSS: Type II transmembrane serine protease
tSNE: t-distributed Stochastic Neighbor Embedding
UIP: Usual interstitial pneumonia
VEGF: Vascular endothelial growth factor
